# High-resolution data on mesoscale dynamics of the Caspian Sea upper layer, obtained in a numerical reconstruction

**DOI:** 10.1016/j.dib.2020.105368

**Published:** 2020-03-23

**Authors:** Gleb S. Dyakonov, Rashit A. Ibrayev

**Affiliations:** a Shirshov Institute of Oceanology, Russian Academy of Sciences, Moscow, Russia; b Marchuk Institute of Numerical Mathematics, Russian Academy of Sciences, Moscow, Russia; c Moscow Institute of Physics and Technology (National Research University), Dolgoprudny, Russia

**Keywords:** Caspian sea, Ocean mesoscale dynamics, Numerical modeling, Sea active layer circulation, Sea circulation reanalysis

## Abstract

Mesoscale dynamics accounts for a significant part of the ocean kinetic energy and plays a crucial role in its mixing. The presented data were generated by numerical simulation and reveal mesoscale patterns of circulation of the upper 15-m layer of the Caspian Sea. The currents in 2003–2005 are reconstructed in an eddy-resolving ocean general circulation model SZ-COMPAS using a realistic forcing. The data arrays have a very high resolution: ∼2 km in space and 4 hours in time. This is sufficient to resolve most of mesoscale features of the sea dynamics as well as a wide range of their temporal spectrum, including inertial oscillations (with period ∼18 hours), synoptic and seasonal variability. The dataset includes: (1) raw model data on the velocity vector fields on three top-layer horizons (surface, 7 m, and 15 m) in 2003, (2) the same fields averaged over every month in 2003–2005, and (3) two video-files visualizing surface currents in 2003–2005 in which sea salinity is used to trace dynamical structures. The data can be used by marine researchers to explore the Caspian Sea dynamics and its impact on the sea biogeochemical condition.

**Table:** Specifications Table

Subject	Earth and Planetary Sciences::Oceanography
Specific subject area	Ocean dynamics
Type of data	Video Binary data (zipped NetCDF file)
How data were acquired	The data were acquired in a numerical simulation of the Caspian Sea hydro- and thermodynamics using an eddy-resolving model SZ-COMPAS
Data format	Raw
Parameters for data collection	Model: SZ-COMPAS; Experiment id: 245; Model resolution: ∼2 km in space, 2–30 m in the vertical, 1 min in time; Bottom topography: ETOPO1 dataset; Initial condition: 3-year spin-up from climatic 3D-fields of temperature and salinity; Atmospheric forcing: ERA-Interim reanalysis dataset; Riverine forcing: realistic discharge of five major rivers.
Description of data collection	The model SZ-COMPAS was run for the period of 2003–2005 with 4-hourly output of velocity and salinity fields in the upper sea layer.
Data source location	Institutions: Marchuk Institute of Numerical Mathematics of RAS, Shirshov Institute of Oceanology of RAS
Data accessibility	Repository name: German National Library of Science and Technology (videos), Mendeley Data (NetCDF files) Data identification number: 10.5446/44428 (video 1) 10.5446/44533 (video 2) 10.17632/st2grwnhmv.1 (NetCDF files) Direct URL to data: https://av.tib.eu/media/44428 (video 1) https://av.tib.eu/media/44533 (video 2) https://data.mendeley.com/datasets/st2grwnhmv/1 (NetCDF files)

**Table:** 

**Value of the Data** • The data reveal mesoscale patterns of circulation of the Caspian Sea upper 15-m layer, which is the most active one. The data are a valuable complement to the existing observational knowledgebase on the Caspian Sea dynamics. • The data can be used by marine researchers (physical oceanographers, marine biologists, limnologists) to study the Caspian Sea dynamics and biogeochemistry. • The velocity vector fields can be used as a marine reanalysis dataset for standalone model simulation of biochemical conditions, transport of polluting substances in the Caspian, etc. The videos visualize sea circulation on the surface and can be used to study the details of its mesoscale dynamics.

## 1 Data description

The presented dataset includes 2 on-line videos and 4 zipped NetCDF files. Videos 1 and 2 visualize surface circulation in the Southern and Middle Caspian, respectively. Sea surface salinity (SSS) is used to trace the dynamics of surface waters and identify its mesoscale patterns, e.g. eddies, jets, intrusions, etc. The period covered is 2003–2005 with 4 hours of real time per frame. Data dimension is psu (practical salinity units). Abrupt drops in the salinity fields (seen as “flashes”) are associated with precipitation events. The third basin of the sea, Northern Caspian, is omitted as it is extremely shallow, and, therefore, its dynamics is rather trivial.

Supplementary video related to this article can be found at https://doi.org/10.1016/j.dib.2020.105368

The following are the supplementary data related to this article:

Multimedia component 2 Multimedia component 2

Multimedia component 3 3 Multimedia component 3

**Fig. 1:**
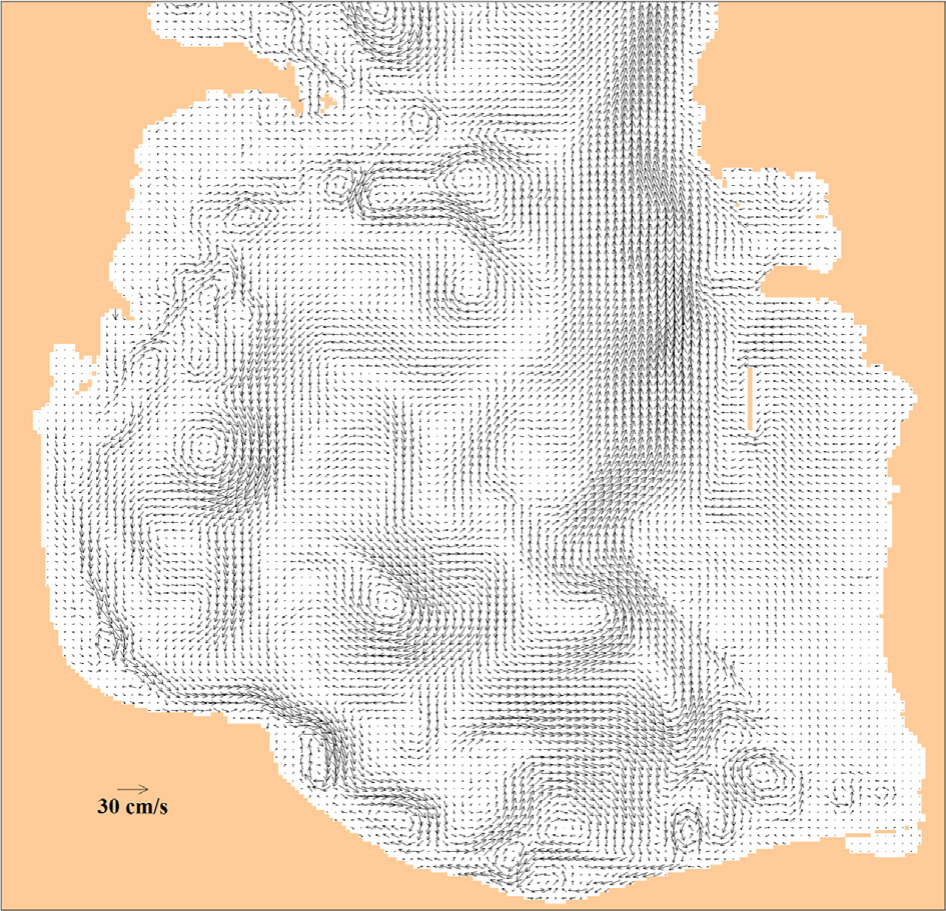
Data sample: model currents in Southern Caspian on 08/29/2003 00:00:00 GMT on 7 m depth (only 25% of all the vectors are plotted to enhance figure readability).

The NetCDF files are: 3 files with instantaneous currents and 3 files with monthly mean currents (suffix “mm” in the file names). Each file corresponds to one of the three horizons (depths): 1 m, 7 m, and 15 m. In the horizontal plane the data are defined on a uniform geographical grid (46.7625–54.2125°E, 36.5092–47.2892°N), dimensions of all of the arrays are 299 by 589. The period covered by instantaneous data is 2003, time step is 4 hours; the first record corresponds to 2003-01-01 04:00:00 GMT. The period covered by monthly mean data is 2003–2005 with one vector field for every month, defined on the last day of the month. All data dimension is cm/s. A sample of the instantaneous currents on the horizon 7 m is given in Fig. 1.

It should be noted that the model describes the upper 30-m layer of the sea in sigma-coordinate, so the data values are actually defined on the 1st, 4th, and 8th sigma-horizons, rather than 1 m, 7 m, and 15 m depths. This means that, in the sea cells with bottom depth less than 30 m, the actual depth of the data nodes is ∼3%, ∼23% and 50% of real water column height, while in the rest of cells (with bottom depth greater than 30 m), the data nodes are located at ∼1 m, ∼7 m, and ∼15 m of depth (± few centimeters depending on the local sea level).

## 2 Experimental design, materials, and methods

Sea currents were calculated using the model described in Ref. [1,2]. It is a three-dimensional ocean general circulation model with a free surface and a variable coastline, adopting hydrostatic, Boussinesq, and water incompressibility approximations. The horizontal resolution is ∼2 km, which is sufficient to describe mesoscale features of water dynamics: in the Caspian Sea the baroclinic Rossby radius of deformation is estimated at 17–22 km in deep regions and 3–8 km on the shelf [3]. The vertical resolution varies from 2 m in the upper layer to 30 m in the abyssal waters. Model time step is 1 min. ETOPO1 dataset [4] is used to set the model bottom relief.

Lateral turbulent viscosity is described by the model via a fourth-order operator with the Smagorinsky parameterization [5] with the minimum dimensionless coefficient C = 2 recommended by the authors. Among the known numerical models of the Caspian Sea, only two [6,7] have a higher resolution (about 1.5 km). However, the model we use has a significantly lower level of dissipation and, therefore, a larger effective resolution, which allows it to reconstruct a wide range of dynamical structures: from large- to mesoscale ones. In order to parameterize the vertical turbulent viscosity, the Munk – Anderson scheme with a maximum coefficient *K*_*m*_ = 10^−3^ m^2^/s is applied.

The boundary conditions on the sea surface are prescribed using the dataset of the European Center for Medium-Range Weather Forecasts (ECMWF) ERA-Interim [8], which has a spatial resolution of 80 km – rather high for global reanalysis but rough relative to the sizes of the Caspian Sea. In order to avoid the so called “land contamination” of the atmospheric fields [9], the creeping sea-fill methodology [10] was applied to properly interpolate the data onto the model grid along the coasts. Riverine forcing is prescribed using realistic data on the discharge of five major rivers: Volga, Ural, Kura, Terek, and Sulak. The sea water outflow into the Kara-Bogaz-Gol Bay is also accounted for. The model had been initialized by three-dimensional climatic temperature and salinity fields [11] and “spun-up” for three years (with the forcing of 2000–2002) until their realistic distribution in the coastal areas established. The T and S fields, which resulted from the “spin-up” run, are used as the initial conditions for the experiment considered.

The Caspian Sea circulation in 2003–2005 has been reconstructed. In 2003 the near-surface wind fields, the main factor in the formation of currents in the upper sea layer, were rather close to the average climatic ones in all months. This allows us to consider the currents obtained in this year as relatively typical for the sea.
